# Sustained Attention Instability as a Cognitive Biomarker in Chronic Spine Pain: A 90‐second Visual Attention Test

**DOI:** 10.1002/ejp.70289

**Published:** 2026-05-06

**Authors:** Kai‐Uwe Lewandrowski, Kenneth Blum, Morgan Lorio, Colin Hanna, Alexander P. L. Lewandrowski, Thomas Lundquist, Rossano Kepler Alvim Fiorelli, Guilherme J. Schmidt, Emmanouil Liodakis, Mark Gold, Juliana J. Schmidt, Eelco Van Duinkerken, Carolina Abramovicz, Andreza P. Maia, Sergio Luis Schmidt

**Affiliations:** ^1^ Department of Orthopedics Hospital Universitário Gaffree Guinle Universidade Federal Do Estado Do Rio de Janeiro Rio de Janeiro Brazil; ^2^ Division of Personalized Pain Research and Education Center for Advanced Spine Care of Southern Arizona Tucson Arizona USA; ^3^ Department of Orthopedic Surgery University of Arizona Tucson Campus Tucson Arizona USA; ^4^ Department of Orthopaedics Fundación Universitaria Sanitas Bogotá Colombia; ^5^ Division of Personalized Genomics The Blum Institute of Neurogenetics & Behavior Austin Texas USA; ^6^ Department of Psychiatry Case Western Reserve University, School of Medicine Cleveland Ohio USA; ^7^ Orlando College of Osteopathic Medicine Winter Garden Florida USA; ^8^ Behavioral Neuropharmacology and Neuroimaging Laboratory on Addictions, Clinical Research Institute on Addictions, Department of Pharmacology and Toxicology Jacobs School of Medicine and Biosciences, State University of New York at Buffalo Buffalo New York USA; ^9^ Department of Psychology State University of New York at Buffalo Buffalo New York USA; ^10^ Department of Biological Sciences Dornsife College of Letters, Arts & Sciences, University of Southern California Los Angeles California USA; ^11^ Brigham Young University Provo Utah USA; ^12^ Neurology Post‐Graduate Program Hospital Universitário Gaffree Guinle, Universidade Federal Do Estado Do Rio de Janeiro Rio de Janeiro Brazil; ^13^ Klinik für Unfall‐, Hand‐ Und Wiederherstellungschirurgie Homburg Germany; ^14^ Center for Sports, Exercise, and Mental Health, Western University of Health Sciences Pomona California USA; ^15^ Department of Psychiatry Washington University School of Medicine St. Louis Missouri USA; ^16^ Department of Pharmacology and Toxicology Jacobs School of Medicine and Biomedical Sciences, University at Buffalo Buffalo New York USA; ^17^ Department of Psychiatry Stanford University School of Medicine Palo Alto California USA

**Keywords:** attention, cognitive dysfunction, executive function, low back pain, neck pain, neuropsychological tests, pain measurement, psychomotor performance, reaction time, spinal diseases

## Abstract

**Background:**

Chronic spine pain is linked to self‐reported cognitive complaints. However, objective markers are lacking. The 90‐s Continuous Visual Attention Test (CVAT) quantifies key attention subdomains: reaction time (RT, alertness), RT variability (VRT, sustained attention), omission errors (focused attention) and commission errors (impulsivity).

**Objective:**

This study aimed to identify which specific attentional subdomains, measured by the CVAT, are impaired in adults with chronic spine pain.

**Methods:**

This prospective case–control study included 84 adults with chronic lumbar/cervical pain (≥ 3 months) and 118 healthy controls. A MANCOVA tested group differences on CVAT variables, controlling for age and sex, followed by Bonferroni‐corrected ANCOVAs. To isolate cognitive variability from general processing speed, the coefficient of variability (VRT/RT) was also analysed. Logistic regression assessed the predictive power of CVAT indices for pain status.

**Results:**

The MANCOVA showed a significant group effect (Pillai's Trace = 0.46, *F*(4,195) = 41.43, *p* < 0.001). Patients exhibited impairments in all measures *p* < 0.001 (*η* ^2^ = 0.093–0.44). The VRT deficit persisted when using VRT/RT. Logistic regression identified VRT as the strongest predictor of chronic spine pain (*χ* ^2^(1) = 147.53, *p* < 0.001), correctly classifying 86.6% of participants. This finding remained when using VRT/RT (*χ* ^2^(1) = 130.55, *p* < 0.001; 82.7% accuracy).

**Conclusions:**

Patients with chronic spine pain demonstrate attentional deficits, with sustained attention instability (VRT and VRT/RT) as the most robust marker. The CVAT detects this impairment, offering a practical tool for clinical assessment to inform treatment and monitor cognitive function in pain management.

**Level of Evidence:**

III (prospective case–control).

**Significance Statement:**

A brief, 90‐s computerized attention test provides an objective, clinic‐ready screen for sustained‐attention instability in spine pain patients. Identifying cognitive vulnerability at the point of care can inform perioperative counselling, driving/work‐safety guidance and rehabilitation planning, and it may help monitor treatment response alongside pain metrics, offering a noninvasive, nonpharmacologic complement to standard pain assessment.

## 1 Introduction

Chronic spine pain is a leading cause of global disability, imposing a substantial personal, societal and economic burden (Hoy et al. 2014; Juniper et al. 2009; Ravindra et al. 2018; Woolf and Pfleger 2003). While clinical management has traditionally focused on nociceptive and structural pathology (Morlion 2013), a growing body of evidence indicates that chronic pain is associated with significant cognitive alterations (Berryman et al. 2014; Higgins et al. 2017; Melkumova et al. 2010; Solberg Nes et al. 2009).

However, identifying cognitive impairments in pain populations remains challenging (Berryman et al. 2013; Eccleston 1995; Khera and Rangasamy 2021). In this context, the assessment of attention and its subdomains is critical as it underlies other cognitive domains, such as memory and executive function. Reaction‐time‐based paradigms have consistently proven sensitive to disruptions in attentional processing in chronic pain (Abudoush et al. 2023; Attridge et al. 2016; Moore et al. 2012; Steinborn et al. 2015; Torkamani‐Azar et al. 2020). However, these deficits in spinal pain populations require clearer characterization, and many existing protocols are too lengthy for routine clinical use (Attridge et al. 2016).

The Continuous Visual Attention Test (CVAT) is a Go/No‐Go paradigm that efficiently quantifies four key attentional subdomains within a 15‐min task: mean reaction time (alertness), variability of reaction time (sustained attention), omission errors (focused attention) and commission errors (response inhibition) (Abramovicz et al. 2025; Bezerra et al. 2024; Fernandes et al. 2023; Simões et al. 2017). This full‐length version has successfully detected attentional deficits in chronic pain and shown that reaction time variability predicts pharmacological treatment response in fibromyalgia (G. Schmidt et al. 2017). As an objective index of attentional stability, the CVAT may offer a more sensitive and an early signal of treatment response compared to traditional patient‐reported outcomes (e.g., visual analogue scales or disability indices), which are vulnerable to recall bias and linguistic or cultural influences (Fairbank and Pynsent 2000). However, its 15‐min duration limits clinical feasibility.

To overcome this, an ultra‐brief, 90‐s version of the CVAT was developed. This abbreviated version demonstrates strong correlations with the full‐length test across all subdomains, with particularly high agreement for reaction time variability (J. Schmidt et al. 2024), and has been validated in diverse clinical settings (e.g., Filho et al. 2025, G. Schmidt et al. 2020).

Despite the high prevalence of spine pain and its recognized cognitive sequelae, the ultra‐brief CVAT has not been systematically validated in this specific population. This study aimed to assess the clinical utility of the 90‐s CVAT in patients with chronic spine pain by comparing their performance to pain‐free controls. We hypothesized that patients would exhibit specific deficits in sustained attention, as reflected by increased reaction time variability.

## 2 Materials and Methods

### 2.1 Study Design

**FIGURE 1:**
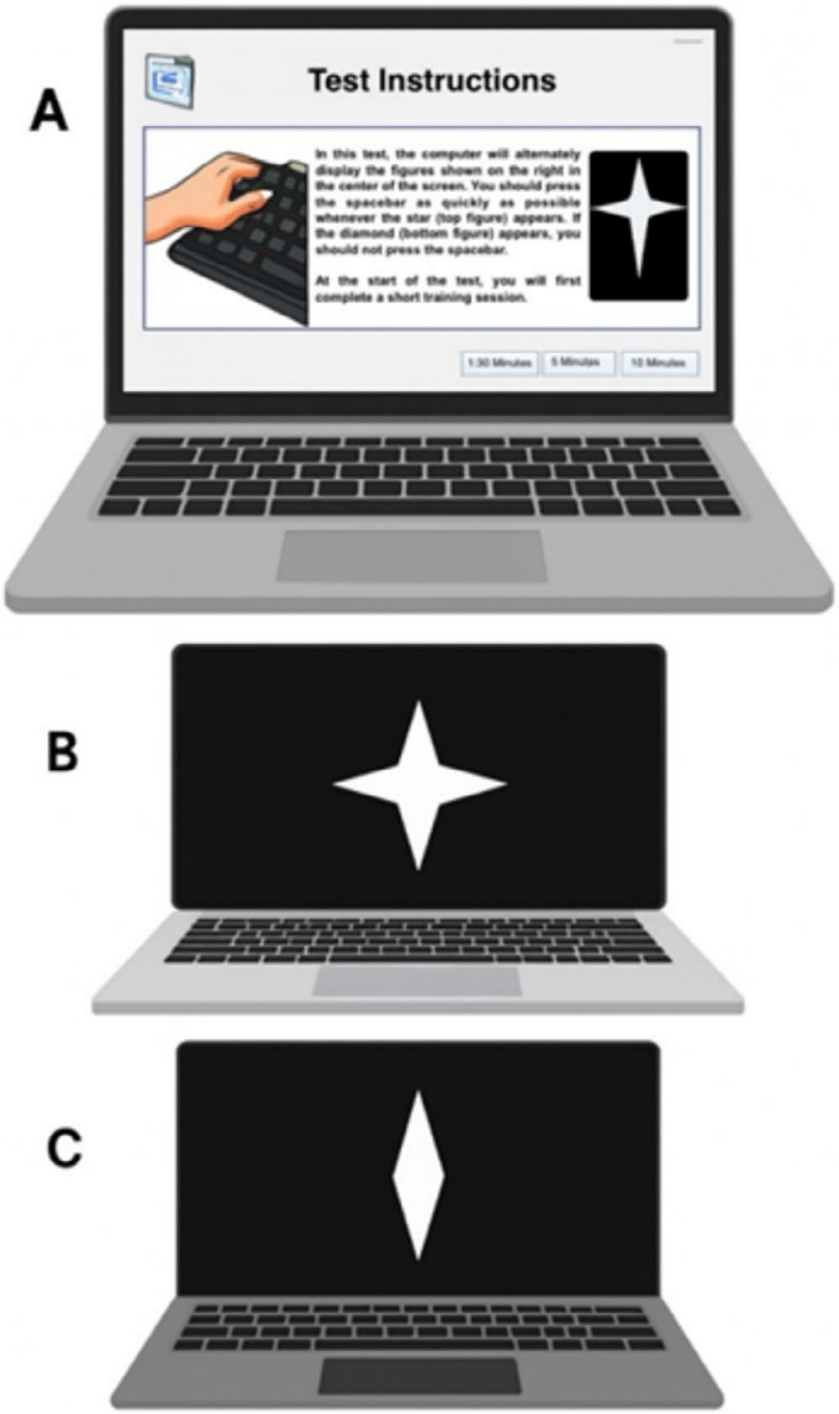
Continuous Visual Attention Test. (A) After on‐screen, interviewer‐read instructions and a brief practice, the task begins. (B) A target (star) appears centrally for 250 ms—participants press the spacebar as quickly as possible with the dominant hand. (C) A non‐target (diamond) also appears for 250 ms—responses are withheld. The session consists of one block of 90 trials with a fixed 1 s inter‐stimulus interval, lasting 1.5 min. Outcomes: Omission errors, commission errors, mean reaction time and variability of reaction time. The CVAT is available to registered psychologists in English, Portuguese and Spanish.

**FIGURE 2:**
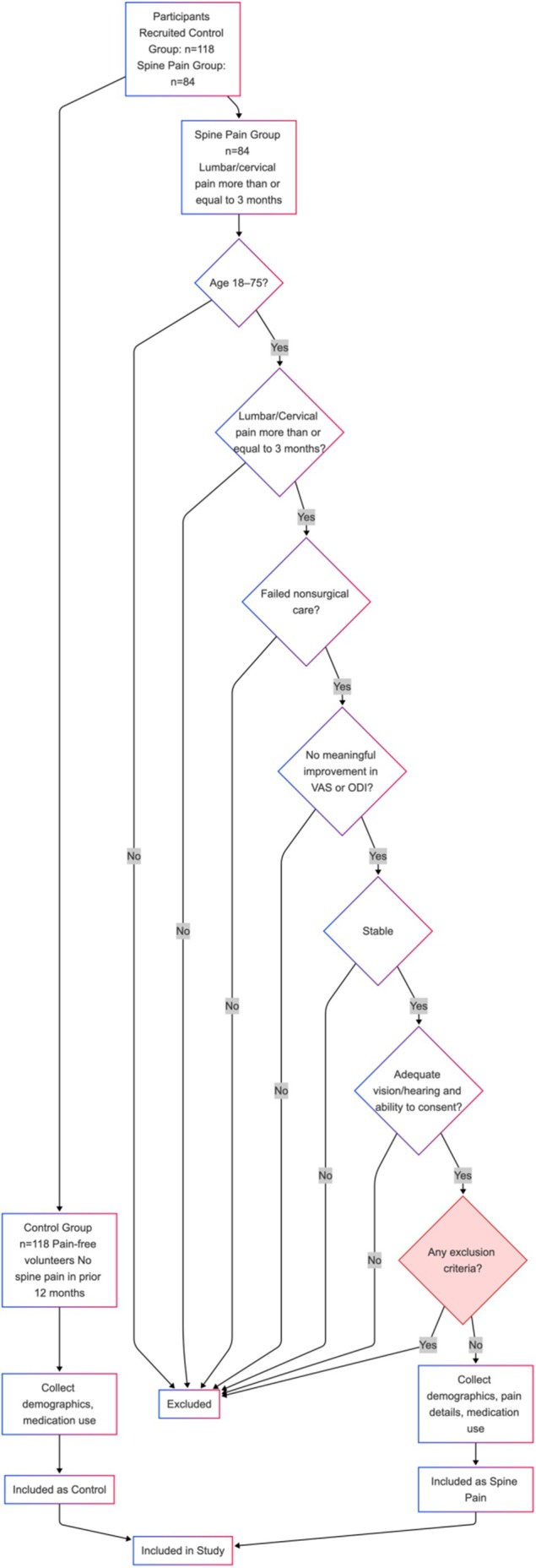
CONSORT‐style flow diagram of study enrollment. A total of 118 controls and 84 spine‐pain patients were screened.

This was a prospective, case–control study designed to evaluate the performance of the 90‐s CVAT in patients with spine pain compared with pain‐free controls (Figure 1; Figure 2). The study protocol was approved by the institutional review board (IRB approval number BB2510KL‐132), and all participants provided written informed consent prior to enrollment.

### 2.2 Participants

Two groups were recruited: spine‐pain group patients presenting with lumbar and/or cervical pain of at least 3 months duration, and a control group, composed of pain‐free volunteers with no history of spine pain within the prior 12 months (Figure 2).

Patients with spine pain were recruited from the Center for Advanced Spine Care of Southern Arizona (Tucson, AZ, USA) and affiliated with private spine‐practice settings in Tucson where standardized CVAT administration and eligibility screening were performed. Pain‐free control participants were recruited from research volunteers and staff at the University of Arizona (Tucson, AZ, USA) and through community‐based recruitment using university‐affiliated networks and local outreach in the Tucson area.

Demographic data (age, sex, education), pain characteristics (numeric rating scale [0–10], pain duration, pain region) and current medication use were collected.

### 2.3 Spine Pain Cohort: Inclusion Criteria

1. Age: 18–75 years.
2. Diagnosis and duration: lumbar and/or cervical spine pain for at least 3 months.
3. Failed comprehensive non‐surgical care: documented lack of clinically meaningful improvement after at least 3 months of non‐surgical management that included the following: (a) structured physical therapy and/or supervised exercise with documented adherence; (b) adequate trials of guideline‐concordant analgesics; (c) at least one image‐guided procedure or documented clinical rationale for not performing such a procedure.
4. No meaningful improvement: < 2‐point reduction on 0–10 pain Visual Analog Scale (VAS) and/or < 10‐point Oswestry Disability Index (ODI) decrease over ≥ 12 weeks (when ODI available).
5. VAS: assessment that characterizes an individual's pain severity on a continuous scale, ranging from 0 to 10, where 0 = no pain and 10 = worst imaginable pain (Bousquet et al. 2009).
6. ODI: self‐report measure of disability in individuals with chronic back pain (Saltychev et al. 2017).
7. No new analgesic class initiation or dose escalation within 14 days prior to CVAT (short‐acting rescue analgesics used routinely and as needed for breakthrough pain allowed).
8. Adequate vision and hearing, as well as the ability to provide informed consent.

### 2.4 Spine Pain Cohort: Exclusion Criteria

1. Neurologic disease affecting cognition/attention (e.g., dementia, stroke, moderate–severe TBI, MS, Parkinson's, epilepsy);
2. Active major psychiatric disorder associated with attentional impairment (e.g., major depression, bipolar, schizophrenia) unless in stable remission;
3. Attention‐altering medications within 30 days (e.g., stimulants, benzodiazepines, antipsychotics, chronic opioids, high‐dose antidepressants, pregabalin/gabapentin);
4. Substance use disorder (alcohol or illicit) currently active:
5. Non‐degenerative spine chronic pain syndromes (e.g., fibromyalgia), autoimmune/rheumatologic disease or cancer‐related pain as the primary pain driver;
6. Recent trauma or surgery: spine pain attributable to acute trauma or spine surgery within 6 months;
7. Task limitations: severe motor impairment, uncorrected visual deficits or inability to complete a valid CVAT practice trial;
8. Concurrent investigational therapy for pain within 30 days.

### 2.5 Continuous Visual Attention Test (CVAT) Protocol

The CVAT is a continuous Go/No‐Go paradigm administered on a laptop computer (Figure 2) (J. Schmidt et al. 2024; Simões et al. 2018). The test has a total of 90 trials, 72 correct targets and 18 non‐targets. Participants were seated approximately 50 cm from the monitor and instructed to press the spacebar as quickly as possible with their dominant hand in response to the target stimulus, and to withhold responses to the non‐target. Each participant was allowed to one training session prior to actual testing. Participants were encouraged to continue the task even after making an error (i.e., pressing spacebar during non‐target or failing to press spacebar during target). The CVAT yields four primary outcome variables: mean reaction time for correct trials (RT), corresponding to tonic alertness subdomain during non‐cued trials; variability of reaction time for correct trials (VRT), indicating intraindividual standard deviation of RTs of correct trials (sustained attention); Omission errors (OE) for missed responses to targets (focused attention); Commission errors (CE) during responses to non‐targets (response inhibition). A secondary measure, the coefficient of variability (VRT/RT), was also considered and calculated.

The 15‐min version CVAT explores high and low target frequencies (80% and 20%, respectively) and fast, medium and slow speeds of stimuli presentation (1, 2 and 4 s). As a previous study has demonstrated that the VRT did not depend on target frequency, speed of stimuli presentation or time duration throughout the 15 min of the task in healthy adults, its 90‐s version exclusively utilizes a high target frequency and fast speed of stimuli presentation. The stimulus duration on‐screen is 250 ms for the 15‐min and 90‐s CVATs. Both versions have a high correlation and significant agreement (J. Schmidt et al. 2024). The 90‐s CVAT has been previously studied in diverse clinical settings (do Carmo Filho et al. 2022; Filho et al. 2025; Gjorup et al. 2026; Taboada Gjorup et al. 2023; G. Schmidt et al. 2020; S. Schmidt et al. 2021, 2022).

### 2.6 Statistical Analysis

All analyses were performed in SPSS v.27 with two‐tailed α = 0.05. MANCOVAS and respective ANCOVAS were conducted to perform comparisons between the two groups using age and sex as covariates. The comparison was made between participants with and without spine pain (patients = spine pain; controls = no pain) on performance measures from the continuous attention test: number of OE, number of CE, mean RT and VRT for correct trials. Prior to modelling, assumptions were assessed as follows: normality via skew/kurtosis and Q–Q plots; homogeneity of covariance matrices via Box's M; and homogeneity of error variances via Levene's test. When assumptions were violated, Pillai's Trace was used as the primary multivariate test statistic due to its robustness. To verify whether variability effects were independent of overall response speed, we repeated the MANCOVA with the coefficient of variability (VRT/RT) in place of VRT.

If the multivariate tests were significant, we conducted Bonferroni‐adjusted ANCOVAs for each CVAT outcome (RT, VRT, OE and CE). For interpretability, we report estimated marginal means with adjusted pairwise contrasts, partial *η* ^2^ for ANCOVAs, and Cohen's *d* for between‐group differences.

To examine group differences in attentional performance, non‐parametric analyses were also conducted using the Mann–Whitney *U* test. This test was chosen as a confirmatory analysis of the group mean differences because the dependent variables showed non‐normal distributions. The comparison was made between participants with and without spine pain (patients = spine pain; controls = no pain) on performance measures from the continuous attention test: number of OE, number of CE, average RT, VRT and the coefficient of variability (VRT/RT).

A multivariate binary logistic regression was performed to examine whether sex, age and performance measures from the CVAT (OE, CE, RT and VRT) predicted the presence of spine pain (0 = no pain; 1 = spine pain). A forward stepwise logistic regression was then conducted using the same predictors. Then, logistic regressions were conducted, replacing the RT and VRT variables with the coefficient of variability (VRT/RT), which represents VRT independently of mean RT. Here, odds ratio (OR) was interpreted as an effect size measure of association between the CVAT variables (CE, OE, RT, VRT and VRT/RT) and spine pain.

### 2.7 Sample Size & Power Calculations

The investigators pre‐specified 80% power at *α* = 0.05 (two‐sided) as the design target. With 118 controls and 84 patients with a spinal pain syndrome, the achieved operating characteristics differ modestly from the original plan: under a simple two‐sample comparison, the minimum detectable standardized effect size (Cohen's *d*) for the primary endpoint (CVAT's VRT) is 0.47. Incorporating ANCOVA (e.g., adjusting for age and sex) improves efficiency in proportion to the variance explained by covariates (*R* ^2^), reducing the detectable effect to *d* = 0.45 for *R* ^2^ = 0.10, *d* = 0.42 for *R* ^2^ = 0.20 and *d* = 0.40 for *R* ^2^ = 0.30. Cohen's *d* expresses the between‐group difference in units of the outcome's standard deviation, enabling comparisons across studies and scales; the original design targeted detection of *d* = 0.40, that is, an average VRT difference of about 0.4 SD between groups. Thus, with the actual total sample size (*N* = 202), the study maintains ≥ 80% power to detect moderate effects (*d* between 0.40 and 0.47 SD) on the primary attention‐variability outcome, closely aligning with the design goal, particularly when covariate adjustment is used.

## 3 Results

**FIGURE 3:**
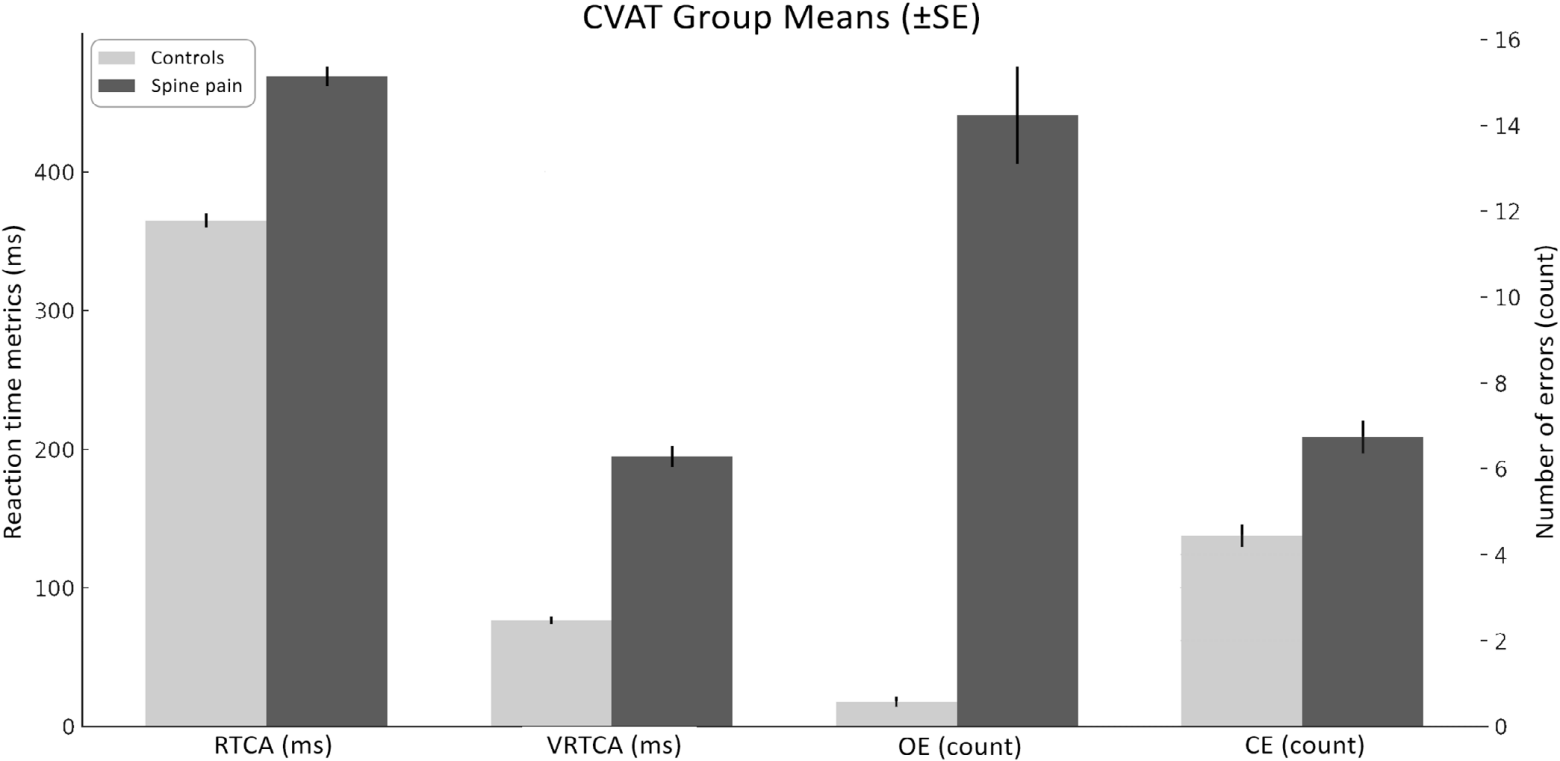
Continuous Visual Performance Test Group Means (±SE). CE, commission errors; OE, omission errors; RT, mean reaction time for correct answers; SE, standard error; VRT, variability of reaction time for correct trials. Bar plots display mean values with standard error of the mean for each attentional variable in asymptomatic controls (*N* = 118) and spine‐pain patients (*N* = 84). Compared with controls, the spine‐pain group demonstrated more OEs and CEs, slower RT and greater VRT, reflecting poorer and less consistent attentional performance. The left *y*‐axis shows RT and VRT (milliseconds), and the right *y*‐axis shows the number of errors (OE, CE). Error bars represent ±SE.

Descriptive statistics for all CVAT variables are presented in Table 1, with corresponding group means visualized in Figure 3. A total of 202 participants were analysed (118 individuals in the control group and 84 in the patient group), with no missing data across any variables. The mean age in the control group was 33.45 ± 13.89 years (range 17–62), compared to 52.80 ± 15.30 years (range 18–75) in the patient group, indicating that patients with spinal pain were generally older. Regarding task performance, the mean number OE was 0.57 ± 1.35 in controls and 14.24 ± 10.41 in patients, with the control group showing a highly peaked distribution (kurtosis = 10.74) and the pain group showing a much wider range (0–35). Similarly, CE averaged 4.45 ± 2.82 in controls and 6.74 ± 3.50 in patients, with both groups demonstrating near‐normal distributions. The average RT was substantially faster in controls (364.80 ± 53.76 ms) compared with patients (468.87 ± 66.82 ms), indicating slower processing speed among patients with spinal pain. Finally, VRT was also greater in patients (194.65 ± 70.22 ms) than in controls (76.47 ± 30.29 ms), reflecting reduced attentional consistency in the pain group. This pattern was further supported by the VRT/RT, which was 0.206 ± 0.065 in controls and 0.408 ± 0.129 in patients. Across all attentional performance measures, patients with a spinal pain syndrome exhibited higher error rates, slower responses and greater intra‐individual variability than the control group without pain, indicating significant impairments in sustained attention and response control associated with chronic spinal pain (Figure 3).

**TABLE 1:** Descriptive statistics for Continuous Visual Attention Test dependent variables.

Variable	Controls *N* = 118	Patients *N* = 84
Age (years)	33.45 ± 13.89 [17.00–62.00]	52.80 ± 15.30 [18.00–84.00]
Omission errors (OE)	0.57 ± 1.35 [0.00–7.00]	14.24 ± 10.41 [0.00–35.00]
Commission errors (CE)	4.45 ± 2.82 [0.00–12.00]	6.74 ± 3.50 [0.00–14.00]
RT (ms)	364.80 ± 53.76 [266.00–558.00]	468.87 ± 66.82 [297.00–603.00]
VRT (ms)	76.47 ± 30.29 [32.00–151.00]	194.65 ± 70.22 [40.00–314.00]
VRT/RT (ratio)	0.206 ± 0.065 [0.095–0.424]	0.408 ± 0.129 [0.120–0.623]

Abbreviations: CE, commission errors; ms, milliseconds; OE, omission errors; RT, mean reaction time for correct trials; VRT, variability of reaction time for correct trials; VRT/RT, coefficient of variability (variability of reaction time normalized by mean reaction time).

### 3.1 Results for the Whole Sample

The omnibus MANCOVA demonstrated significant group differences in attentional performance between spine‐pain patients and controls, Pillai's Trace = 0.46, *F*(4,195) = 41.43 *p* < 0.001, partial *η* ^2^ = 0.46. Age also exerted a significant multivariate effect, Pillai's Trace = 0.11, *F*(4,195) = 0.67, *p* < 0.001, partial *η* ^2^ = 0.11, whereas gender did not. Follow‐up ANCOVAs clarified the specific domains contributing to the group effect. Compared with controls, the spine‐pain group exhibited significantly slower RT, *F*(1, 198) = 65.14, *p* < 0.001, partial *η* ^2^ = 0.25; greater VRT, *F*(1, 198) = 154.03, *p* < 0.001, partial *η* ^2^ = 0.44; more OEs, (*F*(1, 198) = 118.82, *p* = 0.001, partial *η* ^2^ = 0.38) and more CEs *F*(1, 198) = 20.23, *p* < 0.001, partial *η* ^2^ = 0.093. Age was independently associated with attentional performance, predicting slower RTs (*F*(1,198) = 23.62, *p* < 0.001, partial *η* ^2^ = 0.11), higher VRT (*F*(1, 198) = 6.62, *p* = 0.011, partial *η* ^2^ = 0.032). Age did not affect OEs and CEs.

Non‐parametric analysis confirmed significant group differences across all attentional variables. Participants with spine pain showed higher values for OEs, RT, VRT and coefficient of variability, indicating poorer and less consistent attentional performance. Specifically, the spine pain group had significantly more OEs (*U* = 801.50, *Z* = −10.75, *p* < 0.001), CEs (*U* = 3054.00, *Z* = −4.67, *p* < 0.001), longer average RTs (*U* = 1193.50, *Z* = −9.19, *p* < 0.001), greater VRT (*U* = 807.50, *Z* = −10.13, *p* < 0.001).

When VRT and RT were replaced by the coefficient of variability, the same pattern of results was observed. The multivariate analyses, based on Pillai's trace, revealed a significant main effect of group (Pillai's Trace = 0.43, *F*(3,196) = 50.07, *p* < 0.001, partial *η* ^2^ = 0.43). However, age did not significantly influence the outcomes in this analysis. Subsequent univariate tests confirmed significant group differences in OEs (*F*(1,198) = 118.82, *p* < 0.001, partial *η* ^2^ = 0.375), CEs (*F*(1,198) = 20.23, *p* < 0.001, partial *η* ^2^ = 0.093) and the coefficient of variability (*F*(1,198) = 126.34, *p* < 0.001, partial *η* ^2^ = 0.39). Non‐parametric analysis confirmed significant group differences for VRT/RT (*U* = 953.50, *Z* = −9.78, *p* < 0.001).

**FIGURE 4:**
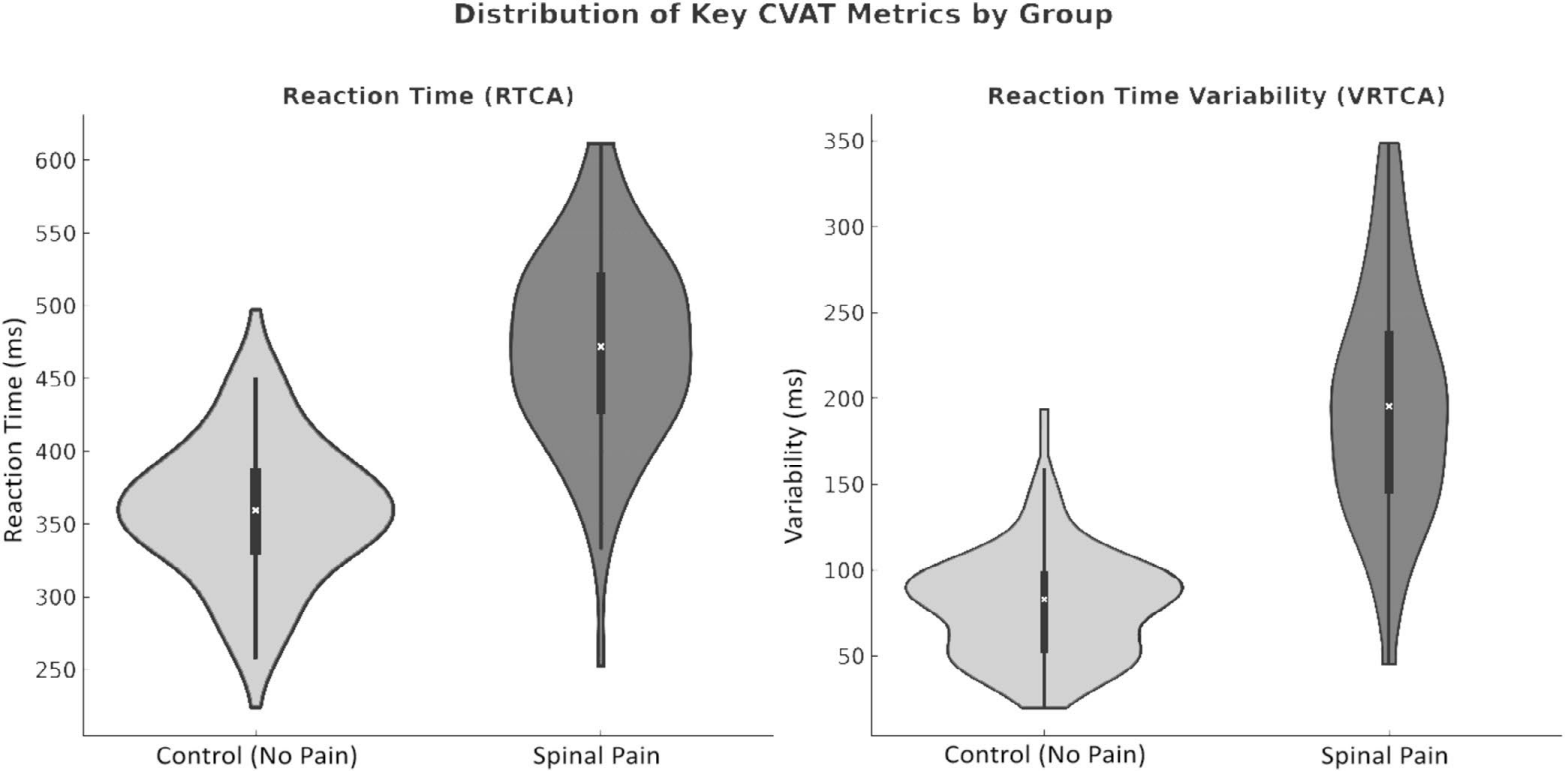
Distribution of Key Continuous Visual Attention Test Metrics by Group: Violin plots display the distribution and central tendency of reaction time for correct trials (RT) and variability of reaction time for correct answers (VRT) for the control and spinal‐pain groups. The wider sections represent a higher density of participants with those values, while the embedded box shows the interquartile range with the median line. Patients with spinal pain exhibited both slower average RTs and markedly greater VRT (*p* < 0.001 for all Mann–Whitney tests), indicating reduced attentional stability and consistency compared with pain‐free controls.

Taking together, these results demonstrate that individuals with spine pain exhibited slower, more variable and less accurate attentional performance compared with pain‐free participants (Figure 4). However, the persistence of group differences in the coefficient of variability indicated that the effect of spine pain on VRT (i.e., sustained attention subdomain) is at least partially independent of RT.

**FIGURE 5:**
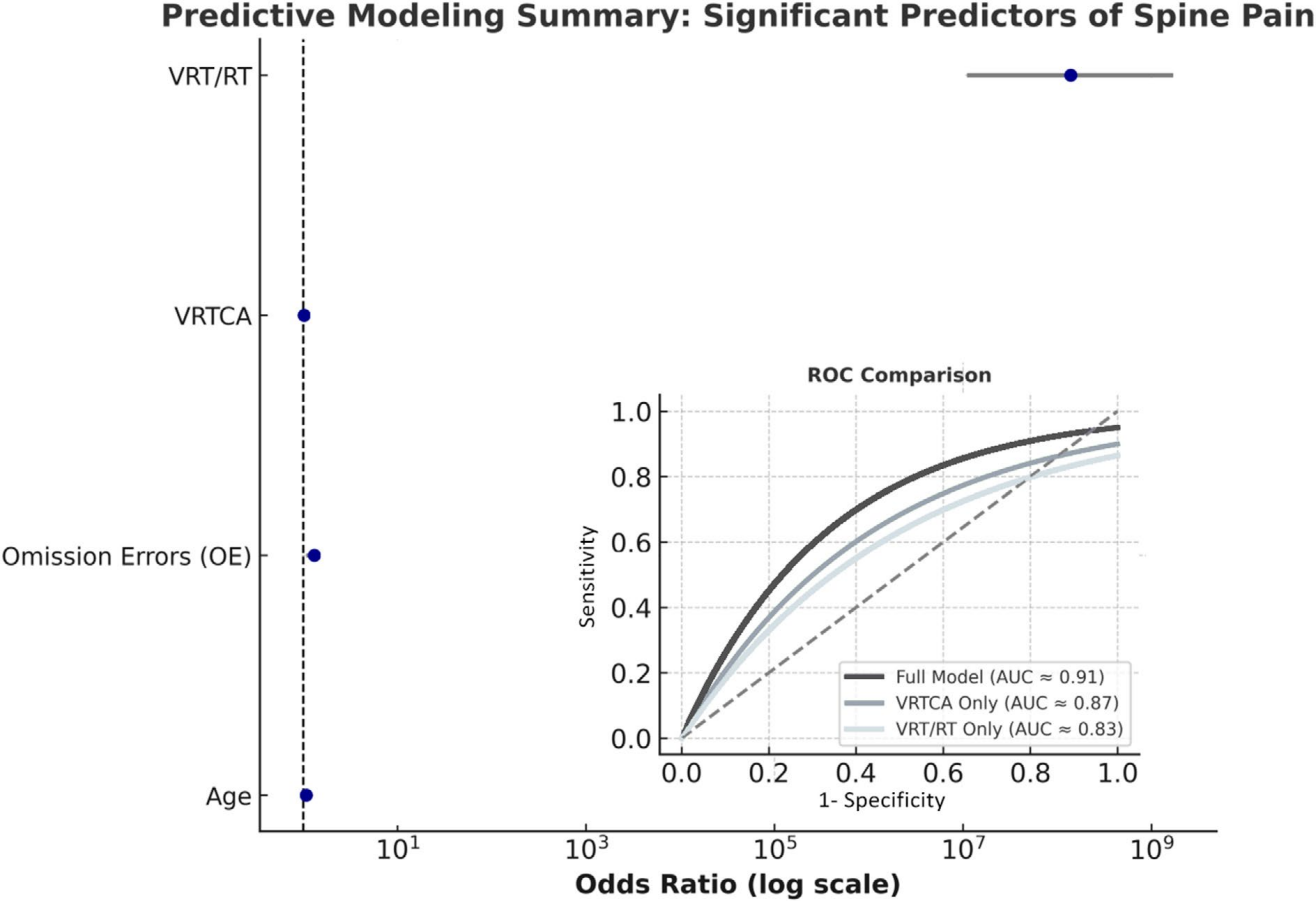
Predictive modelling summary: Forest plot displaying odds ratios (OR, 95% confidence intervals) for significant predictors of spine pain derived from logistic regression analyses. Older age, greater omission errors (OE) and higher variability of reaction time (VRT or VRT/RT) were independently associated with increased odds of reporting spine pain. Odds ratios are plotted on a logarithmic scale; the dashed vertical line indicates the null value (OR = 1). Inset: Receiver operating characteristic (ROC) curves comparing model performance demonstrate excellent discriminative capacity for the full model (AUC = 0.91), with slightly lower accuracy for stepwise models using VRT only (AUC ≈0.87) and VRT/RT only (AUC = 0.83). VRT emerged as the strongest independent predictor of spine pain, reinforcing its clinical relevance as a cognitive biomarker.

Results using binary logistic regressions indicated that when all predictors were entered simultaneously (sex, age, OE, CE, RT, VRT), the model was statistically significant, *χ* ^2^(6) = 174.26, *p* < 0.001, indicating that the predictors reliably distinguished participants with and without spine pain. The model explained between 57.8% (Cox & Snell *R* ^2^) and 77.8% (Nagelkerke *R* ^2^) of the variance in spine pain and correctly classified 90.6% of cases. The Hosmer–Lemeshow test was non‐significant, *χ* ^2^(8) = 3.29, *p* = 0.915, suggesting good model fit. Among the predictors, age (*B* = 0.063, *p* = 0.001, OR = 1.07), number of OEs (*B* = 0.270, *p* = 0.011, OR = 1.31) and VRT (*B* = 0.021, *p* = 0.029, OR = 1.02) were significant (Figure 5). Participants who were older showed greater focus lapses (i.e., more OEs), and had higher VRT were more likely to report spine pain (Table 2).

**TABLE 2:** Predictive modelling: Model fit summary.

Model	*χ* ^2^ (df)	*p*	Cox–Snell *R* ^2^	Nagelkerke *R* ^2^	Accuracy (%)	Hosmer–Lemeshow *χ* ^2^ (df)	HL *p*‐value
All predictors: age, sex, OE, CE, RT, VRT	174.26 (6)	< 0.001	0.578	0.778	90.6	3.29 (8)	0.915
Forward stepwise (VRT only)	147.53 (1)	< 0.001	0.518	0.698	86.6	6.14 (8)	0.632
Forward stepwise (VRT/RT only)	130.55 (1)	< 0.001	0.476	0.641	82.7	9.07 (8)	0.337

Abbreviations: CE, commission errors; ms, milliseconds; OE, omission errors; RT, mean reaction time for correct trials; VRT, variability of reaction time for correct trials; VRT/RT, coefficient of variability (variability of reaction time normalized by mean reaction time).

In the first forward stepwise logistic regression, only VRT entered the model in the first step as a significant predictor of spine pain. The model was statistically significant, *χ* ^2^(1) = 147.53, *p* < 0.001, indicating that VRT reliably differentiated participants with and without spine pain. The model explained between 51.8% (Cox & Snell *R* ^2^) and 69.8% (Nagelkerke *R* ^2^) of the variance and correctly classified 86.6% of cases. Goodness of fit was confirmed by a non‐significant Hosmer–Lemeshow test, *χ* ^2^(8) = 6.14, *p* = 0.632. VRT was positively associated with spine pain (*B* = 0.039, SE = 0.005, Wald = 49.89, *p* < 0.001, OR = 1.039).

Logistic regression performed substituting the separate RT and VRT variables with their ratio (VRT/RT) indicated that the ratio alone entered at Step 1 and significantly predicted the presence of spine pain, *χ* ^2^(1) = 130.55, *p* < 0.001. The model explained between 47.6% (Cox & Snell *R* ^2^) and 64.1% (Nagelkerke *R* ^2^) of the variance and correctly classified 82.7% of participants. The model utilizing VRT/RT as the sole predictor demonstrated substantial explanatory capacity according to these pseudo‐*R* ^2^ metrics and presented adequate fit (Hosmer–Lemeshow *χ* ^2^(8) = 9.07, *p* = 0.337). The VRT/RT ratio was a strong positive predictor of spine pain (*B* = 18.75, SE = 2.59, Wald = 52.52, *p* < 0.001, OR = exp.(18.75 × 0.01) = 1.206 per 0.01‐unit increase in VRT/RT), indicating that individuals with greater relative variability in reaction times were much more likely to report spine pain.

In summary, in both forward stepwise analyses, measures of reaction time variability (either absolute, VRT or relative, VRT/RT) emerged as the most robust single predictor of spine pain. Taking together these findings suggest that attentional instability—manifested by inconsistent RTs—plays a central role in distinguishing individuals with spine pain from those without pain (Table 3).

**TABLE 3:** Predictive modelling: Significant predictors.

Model	Predictor	*B*	SE	Wald	*p*	OR
All predictors	Age (years)	0.063			0.001	1.07
All predictors	Omission errors (OE)	0.27			0.011	1.31
All predictors	VRT (ms)	0.021			0.029	1.02
Forward stepwise (VRT only)	VRT (ms)	0.039	0.005	49.89	< 0.001	1.039
Forward stepwise (VRT/RT only)	VRT/RT (ratio)	18.75	2.59	52.52	< 0.001	Exp (18.75 × 0.01) = 1.206

Abbreviations: *B*, unstandardized regression coefficient; CE, commission errors; ms, milliseconds; OE, omission errors; OR, odds ratio (Odds ratio exp.(*B* × 0.01) per 0.01‐unit increase in VRT/RT); *p*, two‐sided *p*‐value; RT, mean reaction time for correct trials; SE, standard error of *B*; VRT, variability of reaction time for correct trials; VRT/RT, coefficient of variability (variability of reaction time normalized by mean reaction time); Wald, Wald *χ* ^2^ test statistic for *B*.

### 3.2 Sensitivity Analysis (Only Including Participants Younger Than 60 Years)

To evaluate the robustness of our findings across the age distribution, we conducted a sensitivity analysis restricting both samples to participants younger than 60 years. First, we included as many participants as possible from the original cohorts (52 patients and 115 controls). Subsequently, a matched‐pairs analysis was conducted, pairing participants from the two groups by age and sex, resulting in 51 pairs (total *N* = 102, 51 patients and 51 controls). Descriptive statistics confirmed the success of the matching procedure, with no significant differences in the distribution of age or sex between the groups in the paired sample.

A MANCOVA was conducted on the sample with all participants younger than 60 years (*N* = 167) to examine the effect of group membership on CE, OE, RT and VRT, while controlling for age and sex. The multivariate test revealed a significant overall effect of group (Pillai's Trace = 0.613, *F*(4, 160) = 63.23, *p* < 0.001, partial *η* ^2^ = 0.613). Follow‐up univariate tests indicated that the spine‐pain group performed significantly worse than the controls on all measures. Specifically, the spine‐pain patients had a substantially higher number of OEs (*F*(1, 163) = 165.09, *p* < 0.001, partial *η* ^2^ = 0.503), more CE (*F*(1, 163) = 21.18, *p* < 0.001, partial *η* ^2^ = 0.115), a slower average RT (*F*(1, 163) = 86.03, *p* < 0.001, partial *η* ^2^ = 0.345) and significantly greater VRT (*F*(1, 163) = 224.67, *p* < 0.001, partial *η* ^2^ = 0.580). Non‐parametric Mann–Whitney *U* tests corroborated these findings (all *p* < 0.001). An identical MANCOVA conducted on the age‐ and sex‐matched sample (*N* = 102) yielded highly consistent results, confirming the robustness of the group differences (Pillai's Trace = 0.617, *F*(4, 97) = 39.07, *p* < 0.001; all univariate tests p < 0.001).

A MANCOVA on all participants younger than 60 years using the coefficient of variability (VRT/RT) in place of the separate RT and VRT measures again showed a strong multivariate effect of group (Pillai's Trace = 0.583, *F*(3, 161) = 74.91, *p* < 0.001, partial *η* ^2^ = 583). Univariate tests confirmed that the VRT/RT ratio was significantly higher in spine‐pain group (*F*(1, 163) = 176.09, *p* < 0.001, partial *η* ^2^ = 0.519), even after controlling for age and sex. This result was replicated in the matched‐pairs sample (*F*(1, 100) = 121.07, *p* < 0.001, partial *η* ^2^ = 0.548).

The binary regression logistic in the sample of subjects younger than 60 years, including all five predictors (age, sex, OE, CE, RT, VRT) was significant (*χ* ^2^(6) = 139.18, *p* < 0.001, Nagelkerke *R* ^2^ = 0.796), correctly classifying 94.0% of cases. However, a forward stepwise procedure to identify the most parsimonious model selected only VRT and OE as significant predictors. This two‐predictor model was highly significant (*χ* ^2^(2) = 137.75, *p* < 0.001, Nagelkerke *R* ^2^ = 0.790) and maintained a high classification accuracy of 94.0%. For every unit increase in VRT, the odds of being in the spine‐pain group increased by 1.030 (*p* = 0.001), and for every additional omitted error, the odds increased by 1.377 (*p* = 0.014). When the analysis was repeated using the VRT/RT ratio instead of the separate RT measures, the results were consistent. The stepwise model again selected VRT/RT and OE as the sole significant predictors (*χ* ^2^(2) = 132.87, *p* < 0.001, Nagelkerke *R* ^2^ = 0.772), with an overall classification rate of 91.6%. Both the VRT/RT ratio (*B* = 11.079, OR = exp.(11.079 × 0.01) = 1.117 per 0.01‐unit increase in VRT/RT, *p* = 0.008) and the number of OEs (OR = 1.487, *p* = 0.003) were strong, independent predictors. These predictive models were successfully cross‐validated in the matched‐pairs sample, where the stepwise procedure again identified the same two‐variable model (VRT and OE) as optimal (*χ* ^2^(2) = 91.39, *p* < 0.001, Nagelkerke *R* ^2^ = 0.789, Classification Accuracy = 91.2%). Here, we only reported Nagelkerke *R* ^2^, as it represents an adjusted version of Cox & Snell *R* ^2^, which re‐scales the statistic to range between 0 and 1, thereby facilitating interpretation and comparability across models (Bewick et al. 2005).

## 4 Discussion

This case–control study demonstrates that patients with chronic spine pain exhibit significant attentional deficits, which are reliably captured by a 90‐s CVAT. The most robust impairment was observed in the sustained attention subdomain, as measured by VRT. This finding persisted after controlling for age and sex and when using the coefficient of variability (VRT/RT), indicating that the attentional instability is not merely an artefact of generalized psychomotor slowing. The primacy of VRT was further underscored by logistic regression, which identified it as the strongest predictor of case status.

### 4.1 Mechanistic Insights

The observed cognitive profile—marked by elevated VRT, slower mean RTs and more OEs is consistent with a deficit in top‐down attentional control. This pattern aligns with established literature implicating functional and structural alterations in chronic pain states. Specifically, chronic pain involves changes within fronto‐parietal networks, including the dorsolateral prefrontal cortex (DLPFC) and posterior parietal cortex, as well as increased connectivity between subcortical nuclei, fronto‐parietal networks and somatomotor networks (Apkarian et al. 2004; Baliki et al. 2011; Zhu et al. 2024). Pain‐related reorganization of these circuits can lead to the reallocation of cognitive resources (Jaffal 2025). Moreover, sleep disturbances are commonly found in different chronic pain etiologies and exacerbate pain symptoms (Ashworth et al. 2010). In accordance, Zerbini et al. (2025) highlighted that sleep disturbances partially mediated the association between attentional deficits and chronic pain. Consequently, central sensitization and pain‐related cortical reorganization may destabilize these networks, resulting in noisier cognitive performance. This provides a plausible neuroanatomical substrate for the behavioural instability recorded with the CVAT.

### 4.2 Clarifying the Age–Attention Relationship

Although the clinical spine‐pain cohort was older than the control group, the magnitude of attentional disruption far exceeds normative ageing expectations. The effect of VRT remained robust when age was included as a covariate, and the results were replicated in a sub‐analysis of participants under 60 years and in an age‐ and sex‐matched sample. This consistently indicates that the findings are driven by pain‐related factors rather than age. Cognitive‐ageing research describes a profile of ‘slower but organized’ processing, in which the aged brain utilizes alternative or additional neural circuits to accomplish the same tasks as the younger brain, such as bilateral hemispheric activation, accumulated a priori experience and knowledge to form more efficient strategies, increased top‐down activation of frontopartietal networks and higher suppression of the default mode network during cognitively demanding tasks (Cabeza 2002; G. Schmidt et al. 2020; Hedden and Gabrieli 2004; Madden et al. 2007; Park and Reuter‐Lorenz 2009; S. Schmidt et al. 2021). In contrast, the pathologic scores in our pain group, expressed by significantly impaired focused attention and sustained attention (i.e., significantly higher OEs, 14.24 ± 10.41 vs. 0.57 ± 1.35 and considerably increased VRTs, 194.65 ± 70.22 ms vs. 76.47 ± 30.29 ms, in patients compared to controls) could represent a ‘slower and noisy’ profile, which the CVAT quantitatively captures. This mirrors the real‐world clinical scenario where older, multimorbid adults experience fluctuating vigilance under nociceptive load (Dagnino and Campos 2022).

### 4.3 Clinical Implications and Utility

**FIGURE 6:**
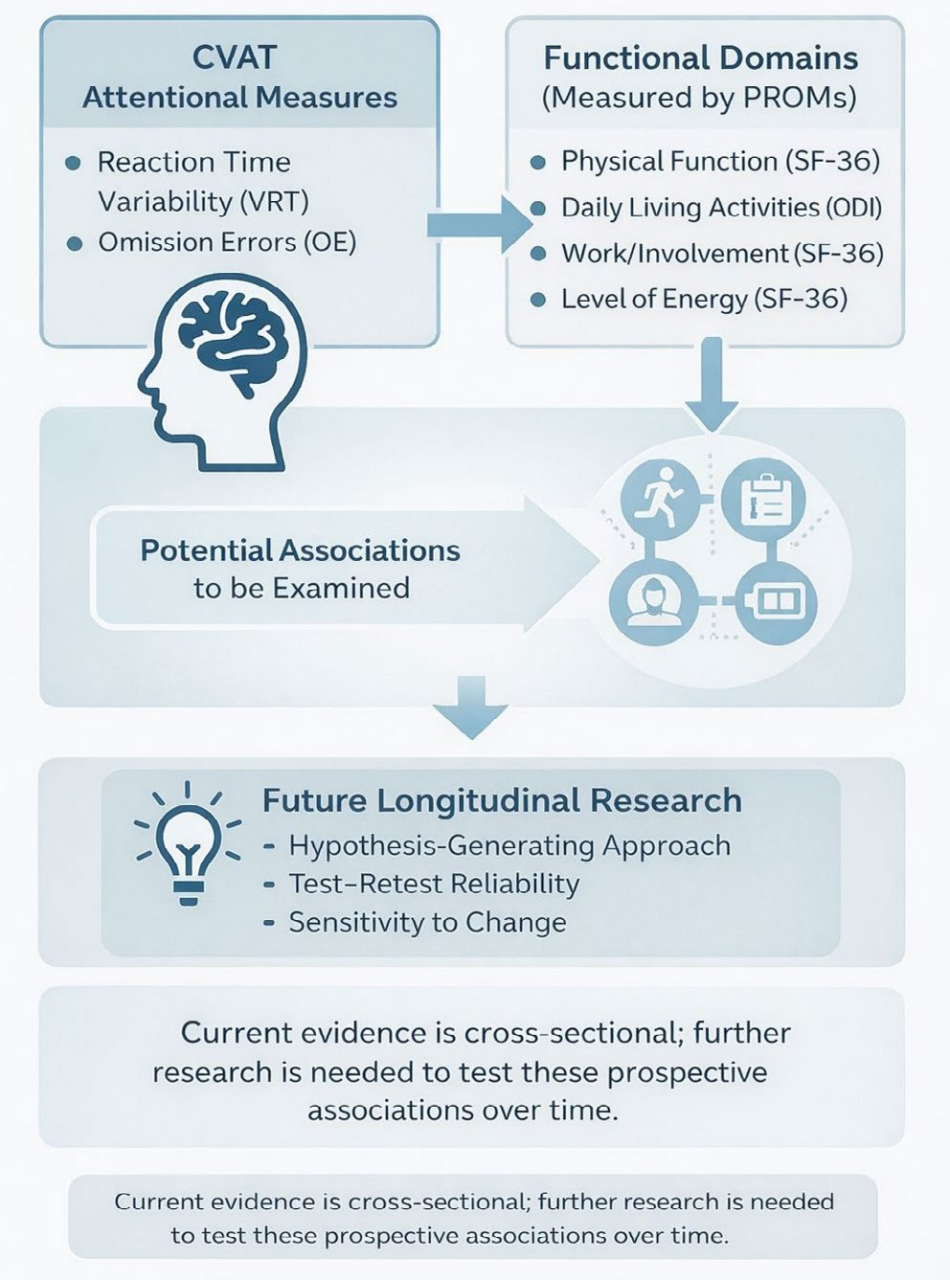
Conceptual framework linking attentional variability to functional domains in chronic spine pain. Performance on the 90‐s Continuous Visual Attention Test (CVAT), particularly variability of reaction time (VRT) and omission errors (OE), may reflect instability in sustained attention. The diagram illustrates hypothesized relationships between attentional variability and domains commonly assessed in patient‐reported outcome measures (e.g., Role‐Physical and Vitality subscales of the 36‐Item Short Form Health Survey ‐SF‐36 and Social Function and the Oswestry Disability Index items‐ODI). These relationships are presented as conceptual and hypothesis‐generating, intended to guide future longitudinal studies examining whether attentional variability is associated with heterogeneity in functional outcomes among patients with chronic spine pain. The current cross‐sectional study does not permit inference regarding prognosis, treatment response or clinical decision‐making.

The CVAT may offer a practical and objective indicator of attentional performance that complements standard patient‐reported outcome measures (PROMs). As summarized in Figure 6, potential future research could explore whether brief attentional assessments with the CVAT provide additional information beyond subjective symptom reports. For example, future studies may investigate whether variability of reaction time is associated with difficulties in cognitively demanding aspects of rehabilitation, adherence to treatment recommendations or functional activities requiring sustained attention. At present, the CVAT should be viewed primarily as a screening tool for detecting objective attentional instability in patients with chronic spine pain, rather than as a rehabilitation instrument or as a standalone tool for guiding clinical or occupational decisions. Broader applications would require additional evidence, including longitudinal studies examining predictive validity, test–retest reliability in clinical populations and sensitivity to change over time. Previous studies have reported minimal practice effects on repeated CVAT administration (Filho et al. 2025; S. Schmidt et al. 2024), which may support its feasibility for repeated measurements in future longitudinal investigations.

Building on these considerations, we propose an Exploratory Framework (Table 4) as a conceptual model to guide future research, which integrates the present finding of attentional instability with known heterogeneity in recovery patterns among patients with spine pain. Specifically, it hypothesizes that elevated VRT and OE at baseline could be associated with slower improvement in outcome domains that depend on sustained attention or mental energy. However, this hypothesis remains speculative and requires validation in longitudinal cohorts, particularly given that many patients with chronic spine pain experience persistent symptoms without full recovery.

**TABLE 4:** Attentional variability and functional domains in chronic spine pain: Relationship between attentional performance (VRT/OE) on the Continuous Visual Attention Test (CVAT) and delayed recovery patterns across SF‐36 and ODI subdomains as a conceptual hypothesis.

Instrument/Domain	Construct measured	Effect of high VRT/OE	Forecasted clinical pattern
SF‐36 Role‐Physical (RP)	Sustained role performance despite pain	Inconsistent task execution, early fatigue	Delayed or smaller RP gain at 6–12 weeks even when pain subsides
SF‐36 Vitality (VT)	Mental energy and cognitive stamina	Attentional fatigue, low arousal	Persistent low VT despite pain relief
SF‐36 Social Function (SF)	Divided‐attention in social interaction	Withdrawal, slowed re‐engagement	Blunted SF recovery even as Bodily Pain improves
ODI (Sitting/Standing/Social Life/Travelling)	Cognitively loaded ADLs needing pacing	Difficulty sustaining posture or planning	Lagging improvement vs. motor‐dominant items (Lifting, Walking)

Abbreviations: ADLs, activities of daily living; CVAT, Continuous Visual Attention Test; ODI, Oswestry Disability Index; OE, omission errors; RP, role‐physical; SF, social function; SF‐36, 36‐Item Short Form Health Survey; VRT, variability of reaction time; VT, vitality.

**FIGURE 7:**
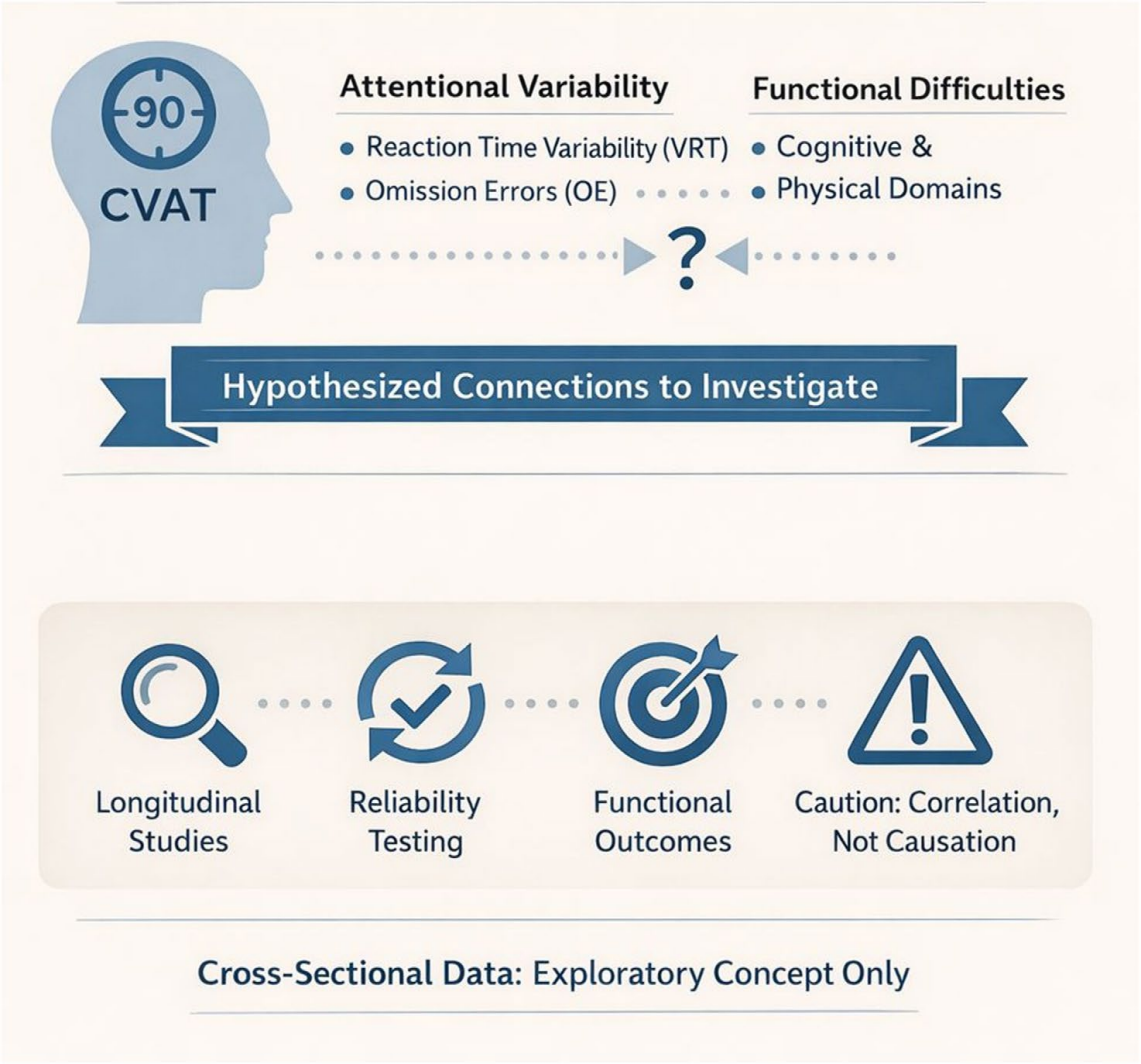
Conceptual hypothesis: attentional variability and functional domains in chronic spine pain. The Continuous Visual Attention Test (CVAT) provides measures of different attentional subdomains, including variability of reaction time (VRT) and omission errors (OE). Increased VRT may reflect instability in sustained attention. The diagram illustrates hypothesized relationships between attentional variability and functional domains commonly assessed in patient‐reported outcome measures, including the Role‐Physical, Vitality and Social Function subscales of the 36‐Item Short Form Health Survey (SF‐36) and items of the Oswestry Disability Index (ODI). These relationships are presented as conceptual and hypothesis‐generating, intended to guide future longitudinal studies examining whether attentional variability is associated with heterogeneity in functional outcomes among patients with chronic spine pain. The current cross‐sectional study does not permit conclusions regarding recovery trajectories, treatment response or prognostic value.

This framework is further illustrated in the Conceptual Hypothesis (Figure 7), which presents attentional stability as a candidate early‐stage marker that could be examined in future prospective studies (Lewandrowski et al. 2025). This framework should be interpreted as hypothesis‐generating rather than predictive, as the present cross‐sectional data do not allow inference regarding recovery trajectories. Longitudinal studies will be required to determine whether brief attentional measures such as the CVAT can meaningfully contribute to understanding variability in clinical outcomes among patients with chronic spine pain.

### 4.4 Strengths, Limitations and Future Directions

Key strengths of this study include its prospective design, use of a validated and ultra‐brief cognitive tool, and a robust analytical approach confirmed with non‐parametric and speed‐controlled analyses.

This study also has limitations. Its cross‐sectional nature precludes causal inference about the relationship between pain and cognition, as the Conceptual Hypothesis (Figure 7) proposes a hypothesis‐generating model to be tested in future longitudinal studies. Furthermore, the study was underpowered for subgroup analyses (e.g., cervical vs. lumbar pain). Moreover, broader applications would require additional investigation, such as test–retest reliability and sensitivity to change. Regarding external validity, the CVAT has been validated in other chronic pain conditions (S. Schmidt et al. 2022). However, normative data specific to chronic spine pain populations is currently unavailable.

Future research should employ longitudinal designs to determine if CVAT metrics, particularly VRT, normalize following successful pain‐relieving interventions and whether baseline performance can predict long‐term functional outcomes. Multimodal studies integrating CVAT with neuroimaging are needed to directly link behavioural variability to DLPFC network integrity in spine pain populations. Finally, future studies are needed to establish condition‐specific normative data and to evaluate generalizability across different chronic pain syndromes.

## 5 Conclusion

The 90‐s CVAT validly identifies sustained‐attention instability in patients with chronic spine pain, with VRT being the most sensitive marker in this cohort. As a rapid and objective cognitive task, the CVAT may complement subjective PROMs by providing an additional indicator of attentional performance. Future longitudinal research is needed to determine whether such measures have prognostic relevance, are sensitive to treatment‐related change, and could contribute to broader clinical assessment frameworks in chronic spine pain.

## Author Contributions

This study was conceived and clinically designed, with project oversight and manuscript leadership provided by Kai‐Uwe Lewandrowski and Sergio Schmidt, under the broader scholarly supervision and editorial guidance of Mark Gold; the theoretical framework linking pain and cognition was developed and interpreted by Kenneth Blum; surgical implications and perioperative pathway considerations were defined by Morgan Lorio; statistical methodology, formal analyses and visualizations were performed by Colin Hanna, Juliana J. Schmidt, Eelco Van Duinkerken, Andreza P. Maia and Sergio Luis Schmidt; data curation, literature synthesis and figure preparation were conducted by Alexander P.L. Lewandrowski; methodological validation and quality assurance were provided by Thomas Lundquist and Sergio Luis Schmidt; integrative interpretation and manuscript synthesis were advanced by Rossano Kepler Alvim Fiorelli; data acquisition, CVAT administration logistics and data management were organized by Kai‐Uwe Lewandrowski, Thomas Lundquist and Guilherme J. Schmidt; patient coordination and regulatory/documentation support were managed by Kai‐Uwe Lewandrowski and Thomas Lundquist; neurocognitive task design, methodological guidance and supervision of cognitive testing were provided by Sergio Schmidt and Eelco Van Duinkerken; neuropsychological assessment design and data curation with drafting and editing contributions were completed by Kai‐Uwe Lewandrowski, Morgan Lorio, Andreza P. Maia and Sergio Schmidt; and neurocognitive/neuropsychiatric expertise with analytical oversight and writing were provided by Sergio Luis Schmidt. Mark Gold and Carolina Abramovicz additionally contributed high‐level supervisory input and editorial refinement across the manuscript. All authors critically reviewed, revised and approved the final manuscript.

## Funding

This work was supported by grants from the Brazilian National Research Council and FAPERJ awarded to Professor Sergio L. Schmidt. No external funding influenced the development, writing or submission of this manuscript. Andreza Maia and Carolina Abramovicz are supported by a CAPES fellowship, J. J. Schmidt and Eelco Van Duinkerken are postdoctoral fellows funded by FAPERJ.

## Disclosure

Use of Artificial Intelligence: The authors of this study did not use AI for the preparation of this manuscript.

Transparency statement: The authors of this paper confirm that the Continuous Visual Attention Test is a non‐commercial research tool. To gain full access to the software, contact the Principal Investigator of this study at sergio.schmidt@unirio.br.

## Conflicts of Interest

The authors declare no conflicts of interest.
